# Anxiety and Depression Among Women Living with HIV: Prevalence and Correlations

**DOI:** 10.2174/1745017902016010059

**Published:** 2020-07-21

**Authors:** Abdilahi Yousuf, Ramli Musa, Muhammad Lokman Md. Isa, Siti Roshaidai Mohd Arifin

**Affiliations:** 1 College of Medicine and Health Sciences, Jijiga University, Jijiga, Ethiopia; 2 Department of Psychiatric, Kulliyah of Medicine, IIUM, Kuantan, Malaysia; 3 Department of Basic Medical Sciences, Kulliyah of Nursing, IIUM, Kuantan, Malaysia; 4 Department of Professional Nursing Studies, Kulliyah of Nursing, IIUM, Kuantan, Malaysia

**Keywords:** Human immune-Deficiency virus, Depression, Anxiety, Comorbidity, CD4 cell, Odds ratio

## Abstract

**Introduction:**

It has been found that HIV positive women are becoming increasingly affected by various illnesses, including Common Mental Disorders (CMDs) such as depression. Such comorbidity escalates the disease progression to the severe stage and commonly hinders treatment adherence. This study determined the prevalence of anxiety and depression amidst women living with HIV.

**Methods:**

Based on a cross-sectional and facility-based study, 357 HIV positive women were recruited using the systematic sampling technique from two public hospitals in Jijiga town, Ethiopia. The Hospital Anxiety and Depression Scale (HADS) was administered for screening, and followed by a pre-tested questionnaire that comprised of Perceived Social Support and HIV stigma.

**Results:**

The results revealed that the prevalence of both anxiety and depression amidst HIV positive women was 28.9% and 32.5%, respectively. In the multivariate analysis, it was discovered that lack of formal education, being divorced, unemployed, and earning a monthly income less than 1400 ETB (37.5 USD) were significantly associated with depression. Women with symptomatic HIV clinical stage III (AOR =2.06, 95% C.I (0.75-5.61), with CD4 cell count below 250 (AOR = 1.14, 95% C.I (0.57-2.28), and with co-infections (AOR= 1.04, 95% C.I (0.40-2.71) also suffered from depression.

**Conclusion:**

The study outcomes show that the prevalence of depression in women with HIV was 32.5%, but they were more likely to be depressed if they were illiterate, divorced, unemployed or had a financial burden. In addition, HIV positive women with less CD4 cell count and in the final clinical stage or suffered from a co-infection were also associated with depressive symptoms. This signifies the public health implications of psychological and cognitive morbidities of the illness among these women with chronic illnesses. Hence, future mental health interventions and HIV care should be integrated with substantial emphasis given to vulnerable groups, including HIV positive women.

## 1 INTRODUCTION

Anxiety and depression are the two most common CMDs that plague society. Depression is characterised with altered mood, low energy, loss of pleasure, and lack of interest that may lead to poor concentration, low self-esteem, poor appetite, guilty feelings, and disturbed sleep. On the other hand, anxiety refers to feelings of fear, distress, and panic disorder that cause phobias and stress. Some anxiety disorders are obsessive-compulsive disorders, generalised and social anxiety disorders, as well as post-traumatic stress disorders. Such mental disorders are a global public health burden, as more than 300 million and 264 million people worldwide are affected by depression and anxiety, respectively [1]. These CMDs are ranked third in contributing to disabilities and infirmities. Depression alone is estimated to represent about 15% of the total global disease burden in 2020 [2].

The recurrence rates of anxiety and depression are two to four-fold amongst HIV positive patients than the rest of the population [3]. These two CMDs, which are a long-standing challenge in the public health sector, are usually unnoticed and remain a potentially risky condition that can affect not only the social aspect of their quality of life, social interactions, and adherence to treatment [4-6], but also their life expectancy [7].

Besides, HIV positive patients with comorbid diseases, such as depression or anxiety, are more likely to acquire viral resistance, and as a consequence, develop poor health outcomes due to low treatment adherence, especially decreased CD4 level [8-12]. Advanced and fatal trajectory has been reported when depression and anxiety occur as comorbid diagnosed in a chronic patient [13].

This may result in substantial public health risk, which raises the need for further studies and effective prevention strategies so as to ascertain better health outcomes and improved quality of life for patients in Sub-Saharan Africa diagnosed with HIV.

The burden of this comorbidity shows the high prevalence of these CMDs among HIV patients than the general population [14]. The magnitude of the problem is amplified when HIV is widespread among those with high vulnerability, stigmatised, and marginalised, such as women, racial or ethnic minority, people with disabilities, or those with a higher level of depression than the rest [15].

So far, depression is highly prevalent in Ethiopia, and it is considered as one of the common psychiatric disorders that affect women as its associates are gender-specific. Some studies conducted in Ethiopia revealed a high prevalence of depression amidst AIDS/HIV patients, whereby 38.94% of the studied participants were depressed [16].

Women diagnosed with HIV have been substantially investigated to address worldwide HIV transmission issues within the biomedical domain. Nevertheless, studies regarding the psychosocial well-being of HIV positive women across developing and developed nations are in scarcity [17].

In developing countries, depression is a serious barrier that must be addressed when discussing HIV prevention as the two diseases are fundamentally linked and aggravate each other. Some studies reported that depressive symptoms and depression reflect the poor adherence to HIV treatment, thus adversely affecting the desired clinical results [18].

Despite the long-reckoned importance of screening depression and managing HIV positive patients across healthcare facilities, their prevalence and risk factors on women have remained untapped. As such, this study exhaustively assessed the prevalence and correlations of anxiety and depression amongst HIV positive women.

## 2 METHODS

This study involved two hospitals based on their comprehensive service provision. In accordance with the national HCT guideline, secondary- and tertiary-level healthcare facilities offer minimum curative and preventive packages. Hence, healthcare facilities were purposively selected for their key service delivery and availability of chronic patients. As such, a cross-sectional study was conducted in antiretroviral treatment clinics of two hospitals located in Jijiga town of eastern Ethiopia in June 2019. The study population comprised of women aged 18 years and above attending HIV treatment service at the two selected hospitals. To estimate the required sample size, a single population proportion formula had been applied. The prevalence of depression (P) from a baseline study in Ethiopia was 38.9% with 5% significant level, Q = 0.05,/2 = 1.96, and 5.0% absolute precision or margin of error tolerance (d = 0.05). The Epi Info software suggested 357 women as the study sample size. The systematic sampling method was applied to select the participants for this study. To determine the sampling interval, those with appointments during the data collection period were divided by the total sample size, wherein the initial point was selected in a random manner.

A trained nurse conducted the interview with women attending the antiretroviral treatment centres using a pretested structured questionnaire. Apart from the socio-demographic profile, their clinical and laboratory investigation results were retrieved as well. Women participants were screened with the HADS tool. This tool is composed of seven questions each for anxiety and depression. Although these 14 questions are interspersed in the questionnaire, they must be scored in a separate manner. A score of 8-10 signifies mild depression, while 11-14 is moderate, and 15-21 indicates severe depression. Besides, the PSS-HIV scale was used to assess the level of perceived support in women with HIV. This 12-item scale has examined women's self-esteem, self-development and belongingness. Moreover, the HIV stigma scale was used to measure the extent of stigma by examining the negative self-image, concerns about public attitude, disclosure concerns and personalized stigma.

The gathered data were analysed using IBM SPSS statistics version 22. Multivariable logistic regression was performed by using forward stepwise logistic regression to identify the independent variables linked with the outcome variable. Variables with p-value 0.005 or less were incorporated into the model. Odds Ratio (OR) and Adjusted Odds Ratio (AOR), along with their Confidence Interval (95%CI), were calculated to identify the strength of the correlations.

Ethical approval of the study was obtained from the International Islamic University of Malaysia Research Ethics Committee (IREC). A permission letter was also obtained from the University's board of review. Written informed consent was collected from the participants after explaining the study aims and other information relevant to this study. The confidentiality and privacy of the participants had been strictly maintained.

## 3 RESULTS

### 3.1 Socio-demographic Characteristics

In total, 357 participants were involved in this study and the mean value of their age was 34.2 years (SD=8.8 years).

Most of the participants were urban dwellers (n=332, 93%) and derived from the Amhara ethnicity (n=130, 36.4%). Among the total participants, 134 (37.5%) were married, and 165 (46.2%) belonged to Islam. Around half of the participants (46.2%) did not attend any formal education, while 129 (36.1%) were unemployed, and 173 (48.5%) reported that their family earned less than 1400 Ethiopian Birr or 37.5 US dollars based on the Ethiopian poverty assessment. Besides, most of them were nulliparous 173 (48.5%). Table **1** tabulates the demographic profile of the participants.

### 3.2 Clinical Characteristics of the Study Participants

The clinical characteristics of the participants were assessed, including clinical staging level, viral load and CD4 level, as well as the comorbidity of the common opportunistic infections.

Out of the 357 participants interviewed, 127 (35.6%) of them were in WHO clinical stage I, and followed by 93 (26.1%) at clinical stage III. As for virological examination, 151 (42.3%) of the participants had a CD4 count above 475 cell/l, and followed by 111 (31.1%) with a CD4 count ranging from 251-475 cell/l. A total of 175 respondents (49.0) complained suffering from at least a common opportunistic infection.

### 3.3 Depression and Anxiety Prevalence

The HADS was used to screen the study participants. The prevalence of depression and anxiety of the participants was 32.5% and 28.9%, respectively, while comorbid prevalence was 22.7%. The scores of anxiety and depression revealed that 18.2% of the participants had symptoms of severe depression, 12.0% experienced moderate depression, and 1.7% suffered from mild depression.

In HADS, a higher rating denotes a higher level of anxiety and depression. For the scales of anxiety and depression, scores under 7 suggest that the participants were not depressed, scores 8-10 reflect mild cases, 11-14 are moderate, and 15-21 display severe cases.

### 3.4 Factors of Depression and Anxiety Among HIV Positive Women

Binary logistic regression was employed in this study to identify the correlations of socio-demographic profiles, as well as clinical and virologic factors, with anxiety and depression. In order to control the influence of a potential third variable, and to avoid non-causal association due to this confounding factor, a step-wise logistic regression was carried out, and the adjusted multivariable was assessed by including factors that were significant in the binary regression analysis.

The outcomes revealed that marital status, education level, occupation, and monthly income of the participants were significantly related to both anxiety and depression. Other socio-demographic variables, such as the age of the participants, residence, ethnicity, faith, and the number of living children, had no association with anxiety and depression.

The clinical characteristics of the participants, such as HIV clinical staging, number of CD4 cell counts, and presence of opportunistic infections, were significantly linked with depression and anxiety, as presented in Tables **2** and **3**, respectively.

In multivariate analysis, the results revealed that divorced women were twice more likely to develop anxiety (AOR = 2.83, 95% CI (0.80-10.1) and four times more likely to acquire depression (AOR = 4.18, 95% CI (1.26-13.8). Women without formal education exhibited more prevalence in anxiety (AOR = 1.71, 95% CI (0.64-4.57) and depression (AOR = 1.45, 95% CI (0.55-3.82) than those with academic background.

The findings displayed a higher prevalence of anxiety (AOR = 4.09, CI (1.56-10.8) and depression (AOR =2.17, 95%CI (0.85-5.56) among unemployed women and they were significantly more prevalent among those who earned less than 1400 ETB a month.

Women with symptomatic HIV clinical stage IV (AOR =3.61, 95% CI (1.07-12.2) for anxiety and clinical stage III (AOR = 2.06, 95% CI (0.75-5.61) for depression, those with CD4 cell count below 250 (AOR=1.62, 95% CI (0.79-3.32) for anxiety and (AOR =1.14, 95% CI (0.57-2.28) for depression, as well as those with an opportunistic infection, were more likely to suffer from anxiety and depression.

Spearman's coefficient test was used to determine the correlation among perceived social support, HIV stigma, and depression.

A significantly negative correlation was found between perceived social support and depression (*r* = *r* = -0.11 p = 0.037) with BCa 95% confidence interval [-0.21 to -0.02] and anxiety (*r* = -0.15 p = 0.005) with BCa 95% confidence interval [-0.26 to -0.04] among the participants.

The lower score of perceived social support among the participants suggested a higher level of anxiety and depression.

Meanwhile, a positive correlation was found between HIV stigma, and depression (*r* = 0.37 p < 0.001) with BCa 95% confidence interval [0.26 to 0.46] and anxiety (r =0.36 p<0.01) with BCa 95% confidence interval [0.24 to 0.46].

This showed that higher scores of HIV stigma among the study participants denoted a higher level of anxiety and depression, as tabulated in Table **3**.

## 4 DISCUSSION

This study had assessed the prevalence and correlations of depression and anxiety among HIV positive women from two selected hospitals in eastern Ethiopia. The work is part of an extended mixed-method study with an emphasis on women living with HIV. This study verifies the results of past research work that reported a higher prevalence in women than men [19-23]. In this study, the symptoms of depression were measured using the HADS screening tool. The other scales used in this study were the PSS-HIV scale to assess social support and HIV stigma. As a result, the prevalence rate of depression among the participants was 32.5%. This is lower when compared to other studies from Sub-Saharan Africa, such as 48% in Kenya [24] and 48.7% in South Africa [25]. This is ascribed to the difference in the study population recruited in these studies, which comprised of women in postpartum/pregnancy with a period of high susceptibility to depression. However, the prevalence of this study is higher when compared to other studies also conducted in developing countries, such as 11% in Malawi [21].

A number of reasons can be attributed to the prevalence of depression in these countries. Firstly, there is a difference in the screening tools used. For instance, studies in Malawi [21], Kenya [24], and South Africa [25] used the EPDS screening tool, whereas a study in Uganda applied the CES-D for screening. The other reasons refer to the methodological variations and the sample size difference.

The comorbid prevalence of anxiety and depression was 22.5%. According to Dell'Osso and Pini [26], the possible clarifications for these comorbid mental illnesses might be that both disorders are likely to share the same phenomena or common symptoms or vulnerabilities. Upon close examination, the findings revealed that the point prevalence of these comorbid illnesses is marginally higher than the institutional-based cross-sectional study conducted on pregnant women in Tanzania (18.1%) [27].

A number of factors have been identified related to the development of anxiety and depression among women living with HIV. For instance, in this study, divorcees were found to be more likely to develop both depression and anxiety. Similar findings have been reported in studies conducted in Ethiopia [28] and Uganda [29], thus revealing that women who were not living with their partner or divorced were at higher risk of acquiring depression. A study conducted in Ukraine by Bailey [30] discovered that non-cohabiting and single mothers were more likely to be depressed. This justifies that broken-up families or insecure marital engagements intensify the risk of developing depressive symptoms. This is commonly observed among abandoned partner, who usually suffers from psychological and emotional disturbances when they miss their loved one(s) or when feeling the burden of family-related workload alone with no or less support.

It was revealed that women who were illiterate, unemployed, and whose monthly household income was less than 1400 Ethiopian Birr were at higher risk for anxiety and depression. The results are consistent with those reported in a study that involved South African HIV positive women, which presented that low-income and unemployment were related to depression [25, 31]. The reason could be that in low-income countries, women are pressured to default academics for poverty-related factors, which later result in their more prominent engagement on domestic work, as well as the lack of access to health education and awareness. This is ascribed to the possible negative interaction between mental disorders (*e.g*., depression) and poverty, primarily because, in principle, people with depression commonly perform poorly in their daily tasks [32]. Moreover, a number of studies carried out in high-income countries revealed that people with social and economic disadvantages are more likely to develop depression [33], indicating the substantial relationships between social adversity, HIV, and depression. Based on the world federation on mental health, women tend to suffer from depression two-fold more than men due to financial issues, lack of formal education, and potential exposure to violence [34].

This study found that women with symptomatic HIV clinical stage IV for anxiety and clinical stage III for depression, CD4 cell count below 250 cells/mm, and opportunistic infection were more likely to suffer from anxiety and depression. Similarly, other studies conducted in the Russian Federation [35], the USA [36], China [9], and Ethiopia [37] reported that a decrease in Cd4 cell counts and an increase in viral load were significantly related to both depression and anxiety. This is attributed to the effect of depression on HIV disease progression by suppressing the immune system, and this immunosuppression, may, in turn, be a predictor for the development of depression among HIV patients [10, 38]. Additionally, the growing evidence suggests the significant role of immunological factors, such as CD4 cells, on the pathophysiology of depression development, particularly among patients with chronic illnesses.

Regarding women's expected support, a significantly negative correlation was reported between perceived social support and depression. Similarly, a study on HIV positive Malawian women indicated that depressive symptoms were associated with decreased perceived social support [39]. In line with this, numerous studies conducted in Sub-Saharan Africa have reported the significant relationship between depressive symptoms and social support [40]. This manifests that social support represents the scope of one's level of satisfaction in relation to the necessity of support, information, and interaction, apart from being a key element for coping with depression. Brittain *et al*. [41] asserted that the substantial moderator between social support and depression was the stigma perceived by women with HIV.

## CONCLUSION

This study found a positive correlation between HIV stigma and depression, which is consistent with previous findings (Benoit *et al*., 2014; Gonzalez *et al*., 2012; Logie *et al*., 2013). Apprehension and depression are attributable to one's expected fear of social acceptance. This emotional disturbance may be related to feelings of not receiving the deserved public attention due to their illness or their depressive circumstances.

Hence, as this study is a cross-sectional one, the possible limitation is the lack of possibility to examine the causal association between social support and stigma with the dependent variable; depression. The other possible limitation is, the interview was not carried out by a psychiatrist who could sub-standardize the quality of the data collection. However, the findings suggest the need for further longitudinal studies in the future by taking into account several potential risk factors.

In conclusion, this study reveals that depression and anxiety are indeed prevalent among women living with HIV. These gender-specific mental health gaps need to be addressed in order to develop effective strategies that enhance the integration of mental health in the HIV care setup.

## Figures and Tables

**Table 1:** Socio-demographic characteristics of women living with HIV attending hospitals, 2019 (n= 357).

**Variable**	**Categories**	**Frequency**	**Percent (%)**
Residence	Urban	332	93.0
-	Rural	25	7.0
Age	18-24	54	15.1
-	25-49	290	81.2
-	>50	13	3.6
Marital Status	Married	134	37.5
-	Single	100	28.0
-	Widowed	77	21.6
-	Divorced	46	12.9
No of living children	Nulliparous/No children	173	48.5
-	1-3	142	39.8
-	4-6	42	11.8
Education	No formal education	166	46.5
-	Formal education	191	53.5
Ethnicity	Amhara	130	36.4
-	Somali	113	31.7
-	Oromo	65	18.2
-	Southern Nations	31	8.7
-	Others	18	5
Faith	Islam	165	46.2
-	Orthodox	151	42.3
-	Catholic	12	3.4
-	Protestant	29	8.1
Occupation	Unemployed	129	36.1
-	Student	89	24.9
-	Self-employed	55	15.4
-	Government employee	84	23.5
Income in ETB	<1400	173	48.5
-	1400-3800	80	22.4
-	>3800	104	29.1

**Table 2:** Multiple regression analysis between anxiety and associated factors among participants attending hospitals, 2019.

**Descriptive**	**HADS-A**		**Odds Ratio [95% CI]**	**Adjusted Odds Ratio (95% CI)**
**Anxiety cases** **n (%)**	**None Anxiety cases** **n (%)**			
**Marital Status**	-	-	-	-
Married	52 (38.8)	82 (61.2)	1	1
Single	23 (23)	77 (77)	2.12 [1.19-3.79]	1.82 [0.56-5.86]
Widowed	19 (24.7)	58 (75.3)	1.94 [1.04-3.61]	1.59 [0.57-4.44]
Divorced	9 (19.6)	37 (80.4)	2.61 [1.16-5.84]	2.83 [0.80-10.1]
**Education**	-	-	-	-
Formal education	67 (35.1)	124 (64.9)	1	1
No formal education	36 (21.7)	130 (78.3)	1.95 [1.22-3.14]	1.71 [0.64-4.57]
**Occupation**	-	-	-	-
Unemployed	28 (21.7)	101 (78.3)	2.04 [1.09-3.80]	4.09 [1.56-10.8]
Student	28 (31.5)	61 (68.5)	0.79 [0.41-1.55]	2.15 [0.93-4.93]
Self-employed	12 (21.8)	43 (78.2)	1.02 [0.48-2.17]	3.44 [1.36-8.73]
Govt employee	35 (41.7)	49 (58.3)	1	1
**Income**	-	-	-	-
<1400	37 (21.5)	135 (78.5)	1.62 [0.93-2.82]	0.97 [0.43-2.18]
1400-3800	34 (42)	47 (58)	0.61 [0.34-1.13]	0.54 [0.26-1.13]
>3800	32 (30.8)	72 (69.2)	1	1
**Clinical staging**	-	-	-	-
Stage I	49 (38.9)	77 (61.1)	1	1
Stage II	23 (28.1)	59 (71.9)	1.63 [0.89-2.98]	1.25 [0.38-4.11]
Stage III	21 (22.6)	72 (77.4)	2.18 [1.19-3.99]	1.59 [0.56-4.55]
Stage IV	10 (5.6)	46 (94.4)	2.93 [1.35-6.34]	3.61 [1.07-12.2]
**CD4 count**	-	-	-	-
<250	18 (18.9)	77 (81.1)	1.66 [0.89-3.11]	1.62 [0.79-3.32]
251-475	42 (37.8).	69 (62.2)	0.62[0.37-1.05]	0.92[0.49-1.69]
>475	43 (28.5)	108 (71.5)	1	1
**OI comorbidity**	-	-	-	-
Yes	41 (23.4)	134 (76.6)	1.69 [1.06-2.69]	1.03 [0.39-2.70]
No	62 (34.1)	120 (65.9)	1	1

**Table 3:** Multiple regression between depression and factors associated with participants attending hospitals, 2019.

**Descriptive**	**HADS-D**		**Odds Ratio [95% CI]**	**Adjusted Odds Ratio (95% CI)**
-	**Depressed n (%)**	**Not Depressed** **n (%)**		
**Marital Status**	-	-	-	-
Married	57 (42.5)	77 (57.5)	1	1
Single	25 (25)	75 (75)	2.12 [1.19-4.25]	2.08 [0.66-6.56]
Separated	23 (29.9)	54 (70.1)	1.44 [0.88-3.02]	1.54 [0.59-4.08]
Divorced	11(23.9)	35 ((76.1)	2.35 [1.15-4.78]	4.18 [1.26-13.8]
**Education**	-	-	-	-
Formal education	68 (39.5)	104 (60.5)	1	1
No formal education	48 (25.9)	137 (74.1)	1.77 [1.22-2.79]	1.45 [0.55-3.82]
**Occupation**	-	-	-	-
Unemployed	31 (24)	98 (76)	2.26 [1.25-4.08]	2.17 [0.85-5.56]
Student	32 (36)	57 (64)	1.27 [0.69-2.35]	1.09 [0.49-2.44]
Self-employed	18 (32.7)	37 (67.3)	1.47 [0.72-2.99]	1.19 [0.50-2.84]
Govt employee	35 (41.7)	49 (58.3)	1	1
**Income**	-	-	-	-
<1400	40 (23.3)	132 (76.7)	1.56 [0.91-2.68]	1.24 [0.56-2.74]
1400-3800	42 (51.9)	39 (48.1)	0.46 [0.25-0.84]	0.49 [0.24-0.98]
>3800	34 (32.7)	70 (67.3)	1	1
**Clinical staging**	-	-	-	-
Stage I	54 (42.5)	73 (57.5)	1	1
Stage II	24 (29.6)	57 (70.4)	1.76 [0.97-3.18]	1.28 [0.40-4.08]
Stage III	23 (24.7)	70 (75.3)	2.25 [1.25-4.05]	2.06 [0.75-5.61]
Stage IV	15 (26.8)	41 (73.2)	2.02 [1.02-4.02]	1.36 [0.45-4.13]
**CD4 count**	-	-	-	-
<250	20 (21.1)	75 (78.9)	1.49 [0.82-2.74]	1.14 [0.57-2.28]
251-475	53 (47.7)	58 (52.3)	0.44 [0.26-0.73]	0.50 [0.28-0.92]
>475	43 (28.5)	108 (71.5)	1	1
**OI comorbidity**	-	-	-	-
Yes	48 (27.4)	127 (72.6)	1.58 [1.01-2.47]	1.04 [0.40-2.71]
No	68 (37.4)	114 (62.6)	1	1

## LIST OF ABBREVIATIONS

AIDS: = Acquired Immune Deficiency Syndrome
ART: = Antiretroviral Therapy
AOR: = Attributed Odds Ratio
CD4: = Cluster of Deferentiation-4
CMD: = Common Mental Disorders
COR: = Crude Odds Ration
HADS: = Hospital Anxiety and Depression Scale
HIV: = Human Immunodeficiency Virus
OI: = Opportunistic Infection
WLHIV: = Women Living with HIV
